# Testing mixing rules for structural and dynamical quantities in multi-component crowded protein solutions

**DOI:** 10.1063/5.0204201

**Published:** 2024-05-29

**Authors:** Alessandro Gulotta, Saskia Bucciarelli, Felix Roosen-Runge, Olaf Holderer, Peter Schurtenberger, Anna Stradner

**Affiliations:** 1 Division for Physical Chemistry, Lund University, Naturvetarvägen 14, 22100 Lund, Sweden; 2 Department of Biomedical Sciences and Biofilms-Research Center for Biointerfaces (BRCB), Faculty of Health and Society, Malmö University, Malmö, Sweden; 3 Jülich Centre for Neutron Science (JCNS) at Heinz Maier-Leibnitz Zentrum (MLZ), Forschungszentrum Jülich GmbH, Garching, Germany; 4 LINXS Institute of Advanced Neutron and X-ray Science, Lund University, Lund, Sweden

## Abstract

Crowding effects significantly influence the phase behavior and the structural and dynamic properties of the concentrated protein mixtures present in the cytoplasm of cells or in the blood serum. This poses enormous difficulties for our theoretical understanding and our ability to predict the behavior of these systems. While the use of course grained colloid-inspired models allows us to reproduce the key physical solution properties of concentrated monodisperse solutions of individual proteins, we lack corresponding theories for complex polydisperse mixtures. Here, we test the applicability of simple mixing rules in order to predict solution properties of protein mixtures. We use binary mixtures of the well-characterized bovine eye lens proteins *α* and *γ_B_* crystallin as model systems. Combining microrheology with static and dynamic scattering techniques and observations of the phase diagram for liquid–liquid phase separation, we show that reasonably accurate descriptions are possible for macroscopic and mesoscopic signatures, while information on the length scale of the individual protein size requires more information on cross-component interaction.

## I. INTRODUCTION

Macromolecular crowding is a ubiquitous and relevant factor in biological fluids such as the cytoplasm and blood serum, expressing the seemingly trivial finding that macromolecules in natural and biotechnological solutions are affected by the presence of other molecules.^1,2^ The kinetic and thermodynamic consequences such as varied reaction rates and equilibria^3^ and lowered solution stability^4,5^ govern largely protein diffusion and interaction, as well as the phase behavior and viscosity of protein solutions. For globular proteins, a colloidal picture has proven highly useful for an in-depth and quantitative understanding of concentrated solutions of proteins, which is of high relevance for understanding of function and misfunction in protein solutions, related in particular, to diseases caused by protein condensation.^6,7^ Particular examples include the liquid–liquid phase behavior,^8–12^ cluster formation^13–17^ as well as the slowing down of self- and cage diffusion,^18–23^ eventually leading to dynamical arrest.^16,24,25^

For pure solutions of one protein, the physical picture and modeling approaches up to rather concentrated solutions are well developed and can be used also to predict properties with qualitative and even reasonably quantitative accuracy. Here, pure solutions also include systems where a protein is dissolved in a complex mixture of different additives such as salts and co-solvents that is then treated as an effective solvent.^12,26^ This situation changes once mixtures of different proteins are involved, as the presence of additional components renders the theoretical treatment more complicated.^27–30^ In addition, computational approaches are more involved and costly, as interactions between larger particles in a bath of smaller ones require the simulation of a large number of particles.^24^ This lack of understanding is very unfortunate, as natural fluids, in general, are multi-component systems and show clear effects of the polydisperse crowding on *inter alia* phase behavior^31,32^ and protein diffusion.^33–35^

In this paper, we systematically and comprehensively explore a well-established binary protein system of *α* and *γ_B_* crystallin protein from the bovine eye lens fluid as a first step toward an approximate understanding of multi-component systems.

The class of crystallin proteins present in the eye lens has been intensively investigated in the past, due to their link to the eye lens function and conditions such as cataract. The motivation for a structural and dynamical characterization of these crystallin solutions is thus threefold: (i) they can serve as a well-defined model system for the general investigation of crowding effects in biological cells; (ii) they are well suited for the study of so-called protein condensation diseases;^6^ and (iii) their dynamic properties and, in particular, the occurrence of an arrest transition have been believed to be the underlying mechanism responsible for the loss of lens flexibility observed in presbyopia.^24,36–39^ Briefly, three distinct protein families are found in the vertebrate eye lens, called *α*, *β*, and *γ* crystallin.^40^ In this work, we focus on two crystallin proteins extracted from the bovine lens: *α* crystallin and *γ_B_* crystallin.

Bovine *α* crystallin, a multi-subunit protein with a molecular weight *Mw* ≈ 800 kDa and an average diameter d≈15 nm, has been successfully described with a polydisperse hard sphere model.^18,24,41,42^ Combining x-ray photon correlation spectroscopy (XPCS), neutron spin echo spectroscopy (NSE), dynamic (DLS) and static (SLS) light scattering, zero-shear viscosity measurements, and molecular dynamics simulations of highly concentrated *α* crystallin solutions, the dynamical arrest transition (i.e., glass transition) has been shown to occur at volume fractions around ϕ≊0.58,^18,19,24^ driven by the so-called caging effect for hard sphere systems.^43,44^

A fundamentally different scenario is observed for the monomeric *γ_B_* crystallin fraction ( Mw≊ 21 kDa, d≊ 4 nm), a subclass of the bovine *γ* crystallins. It has been shown to interact via weak, short-range attractions that lead to a liquid–liquid phase separation of *γ_B_* crystallin solutions upon lowering the temperature.^25,45,46^ Dynamic properties, such as collective diffusion coefficients measured via DLS, thus show a strong dependence on the temperature close to the liquid–liquid coexistence curve (or binodal) due to critical phenomena.^25^ The arrest transition for *γ_B_* crystallin occurs at volume fractions significantly lower than hard spheres^43,47^ due to short-range attractions and patchy interactions.^19^ In fact, interprotein interactions induce the formation of transient networks and/or clusters that slow down the dynamics at relatively low volume fractions, which is further enhanced by the nonspherical monomeric shape.^48^ According to a recent study performed via microrheology measurements, the glass transition of *γ_B_* crystallin occurs at a volume fraction between ϕ≈ 0.27 and 0.31.^49^ Interestingly, the glass transition for the *γ_B_* crystallin shows no temperature dependence in the investigated temperature-range.

A comprehensive characterization of mixtures of crystallin proteins is so far missing. While phase behavior and the underlying protein interactions have been addressed,^29,31,50,51^ dynamical and rheological properties remained uncharacterized, despite their high relevance for a complete understanding of eye function and eye conditions such as presbyopia.

This study attempts to explore simple mixing rules based on the detailed knowledge of the individual crystallins and test whether such models are capable of reproducing the concentration and temperature dependence of key dynamic parameters such as the collective diffusion coefficient and the relative viscosity of crystallin mixtures. For binary mixtures of *α* and *γ_B_* crystallin, we provide a characterization of their (i) phase behavior, (ii) the osmotic compressibility measured by SLS, (iii) structural properties via small angle x-ray scattering (SAXS) (iv) zero-shear viscosities utilizing both DLS- and confocal laser scanning microscopy (CLSM)-based microrheology measurements, and (v) collective diffusion coefficients from DLS and NSE experiments. We then compare the results of the $\alpha \gamma_{B}$ binary mixtures to the theoretical predictions, calculated by appropriately weighted combinations of the two properties from the individual or pure crystallin solutions. Given the large number of individual parameters discussed below, we provide a glossary of the used variables for pure and binary lens crystallin solutions in Table I.

**TABLE I.** Glossary of used variables for pure and mixed solutions.

	$\alpha \gamma_{B}$	*α*-crystallin	*γ_B_*-crystallin
Variable	mixture	in pure solution or mixture	in pure solution or mixture
Weight concentration mg/mL	$c_{\alpha \gamma_{B}}$	$c_{\alpha }$	$c_{\gamma_{B}}$
Volume fraction ϕ	$\phi_{\alpha \gamma_{B}}$	$\phi_{\alpha }$	$\phi_{\gamma_{B}}$
Scattered intensity *I*(*q*)	$I_{\alpha \gamma_{B}}(q)$	$I_{\alpha }(q)$	$I_{\gamma_{B}}(q)$
Form factor: c-normalized *I*(*q*) of			
a dilute solution with *c*_0_; $I^{FF}(q)$	$I_{\alpha \gamma_{B}}^{FF}(q)$	$I_{\alpha }^{FF}(q)$	$I_{\gamma_{B}}^{FF}(q)$
Form factor normalized to 1 at q= 0; *P*(*q*)	⋯	⋯	$P_{\gamma_{B}}(q)$
Structure factor *S*(*q*) of *α* and *γ_B_* taken			
at their effective volume fraction $\phi_{\text{eff}}$	⋯	$S_{\phi_{\alpha ,\text{eff}}}(q)$	$S_{\phi_{\gamma_{B}\text{,eff}}}(q)$
Collective diffusion coefficient in the			
limit q→ 0 (DLS); D(ϕ,q→0)	$D_{\alpha \gamma_{B}}(\phi_{\alpha \gamma_{B}}$)	$D_{\alpha }(\phi_{\alpha }$)	$D_{\gamma_{B}}(\phi_{\gamma_{B}}$)
Free diffusion coefficient in the q→ 0 and			
c→ 0 limit, *D*_0_	$D_{0,\alpha \gamma_{B}}$	$D_{0,\alpha }$	$D_{0,\gamma_{B}}$
Collective diffusion coefficient at finite *q* (NSE): D(ϕ,q)	$D_{\alpha \gamma_{B}}(\phi ,q)$	$D_{\alpha }(\phi ,q)$	$D_{\gamma_{B}}(\phi ,q)$

## II. MIXING RULES

Before discussing specific results, we present the basic idea of mixing rules for the prediction of relevant structural and dynamic properties of multi-component protein solutions by calculating a weighted average of the corresponding quantities of the different single-component systems. While the general idea of mixing rules is simple, the choice of the specific form invokes an underlying physical picture. Previous theoretical and experimental studies have provided evidence that for complex protein mixtures as for example present in cells with their thousands of different proteins, important features such as the location of phase boundaries or the diffusion of individual proteins are primarily determined by the effective volume fraction of the particular protein species.^52,53^ It has been pointed out that overall, specific strong attractions between different protein species in the cell cytosol are largely absent, and they thus interact not unlike an effective hard sphere liquid. As a result, self-diffusion of individual proteins is then for example primarily dependent on the overall protein volume fraction. Phase boundaries for liquid–liquid phase separation (LLPS) or crystallization, on the other hand, depend on the interactions between a particular protein type and thus largely on the effective volume fraction of this protein species.

For our study, we build on these findings and on the known properties of the two lens crystallin proteins investigated. We assume that *α* crystallin interacts with itself and with *γ_B_* crystallin through excluded volume interactions only, whereas *γ_B_* crystallin also experiences a short-range attractive interactions with itself, resulting in LLPS at lower temperatures. As a result, we then construct a series of mixing rules based on the assumption that the behavior of *α* and *γ_B_* crystallin in $\alpha \gamma_{B}$ mixtures can be described as effective single-component solutions with an effective volume fraction that also takes into account the reduced accessible volume due to the presence of another hard sphere-like component. These effective volume fractions $\phi_{\alpha ,\text{eff}}$ and $\phi_{\gamma_{B},\text{eff}}$ can then be expressed as

$$\phi_{\alpha ,\text{eff}}=\frac{\phi_{\alpha }}{1-\phi_{\gamma_{B}}},$$ (1)

and

$$\phi_{\gamma_{B},\text{eff}}=\frac{\phi_{\gamma_{B}}}{1-\phi_{\alpha }},$$ (2)

where $\phi_{\alpha }$ and $\phi_{\gamma_{B}}$ are the actual volume fractions of the two proteins in solution.

### A. Static scattering intensity

In order to illustrate how this picture translates into a mixing rule for static quantities, we start with the description of the static scattering intensity *I*(*q*) as a function of the magnitude of the scattering vector *q* as measured for example in a SAXS experiment. For a binary mixture of hard spheres with in total *N* particles, *I*(*q*) is given by

$$I(q)=N\sum_{i,j=1}^{2}F_{i}(q)F_{j}(q)S_{ij}(q),$$ (3)

where we have omitted some prefactors relating specific molecular properties to the absolute scattering intensity, and where $F_{i}(q)$ and $F_{j}(q)$ are the scattering amplitudes of an individual particle for the two particle species, and $S_{ij}(q)$ the so-called partial structure factors, respectively.^54^

Within the underlying assumption of describing the contributions of each species to a solution property as those from an effective one-component system defined by an effective volume fraction, we thus rewrite the full scattered intensity $I_{\alpha \gamma_{B}}(q)$ of the binary system as a sum of two effective self-terms as follows:

$$I_{\alpha \gamma_{B}}(q)=c_{\alpha }\cdot I_{\alpha }^{\text{FF}}(q)\cdot S_{\phi_{\alpha ,\text{eff}}}(q)+c_{\gamma_{B}}\cdot I_{\gamma_{B}}^{\text{FF}}(q)\cdot S_{\phi_{\gamma_{B},\text{eff}}}(q).$$ (4)

The scattering of the mixed solution is thus described only by an intensity-weighted average of the single-component solutions. Here, $c_{\alpha }$ and $c_{\gamma_{B}}$ are the concentration of the pure crystallins in mg/mL and the total protein concentration $c_{\alpha \gamma_{B}}=c_{\alpha }+c_{\gamma_{B}}$. $I_{\alpha }^{\text{FF}}(q)$ and $I_{\gamma_{B}}^{\text{FF}}(q)$ are the concentration-normalized scattered intensities of a low-concentration, non-interacting solution (i.e*.,* form factor).

$S_{\phi_{\alpha ,\text{eff}}}(q)$ and $S_{\phi_{\gamma_{B},\text{eff}}}(q)$ are the structure factors for the pure components taken at the effective volume fraction, as calculated using Eqs. (1) and (2). With this approach, we thus assume that the effect of mutual interactions between *α* and *γ_B_* crystallins can be represented by an increased excluded volume effect in the interactions between the individual crystallins (*i.e., α* - *α* and *γ_B_* - *γ_B_*).

### B. Diffusion coefficients

While mixing rules for static scattering can thus be assumed as simple additive quantities of individual components, dynamic quantities show an additional complexity: in addition to the direct excluded volume interaction, hydrodynamic interactions that depend mainly on the total volume fraction also affect the dynamics. To take the hydrodynamic slowing down into account, we base the mixing rule on the well-known relation for collective colloidal diffusion,^55^

$$D(q)=D_{0}\frac{H(q)}{S(q)},$$ (5)

where the free diffusion coefficient *D*_0_ is modulated by both the static structure factor *S*(*q*) and the hydrodynamic function *H*(*q*) to obtain the lengthscale-dependent diffusion function *D*(*q*).

#### 1. Gradient diffusion from dynamic light scattering

In DLS experiments, the characteristic length scale 2π/q probed is much larger than the protein diameter *d*. DLS therefore probes the so-called gradient diffusion coefficient given by D(qd→0). Thus, we use the established relation for *H*(0) in the limit of qd→0 to correct for hydrodynamic effects

$$H(0)=\frac{U_{s}}{U_{0}}={\left(1-\phi \right)}^{5.4},$$ (6)

where $U_{s}/U_{0}$ represents the concentration-dependent short-time sedimentation velocity.^56^ For *D*_0_, we calculate the intensity average from the individual diffusion coefficients $D_{0,\alpha }$ and $D_{0,\gamma_{B}}$ at their effective volume fractions. We thus obtain the effective average free diffusion coefficient $D_{0,\alpha \gamma_{B}}$ from

$$D_{0,\alpha \gamma_{B}}=\frac{c_{\alpha }\cdot I_{\alpha }^{FF}(0)\cdot D_{0,\alpha }+c_{\gamma_{B}}\cdot I_{\gamma_{B}}^{FF}(0)\cdot D_{0,\gamma_{B}}}{c_{\alpha }\cdot I_{\alpha }^{FF}(0)+c_{\gamma_{B}}\cdot I_{\gamma_{B}}^{FF}(0)}.$$ (7)

The corresponding effective average $S^{\text{eff}}(q)$ in the limit of qd→0 is calculated using

$$S^{\text{eff}}(0)=\frac{c_{\alpha }\cdot I_{\alpha }^{FF}(0)\cdot S_{\phi_{\alpha \text{,eff}}}(0)+c_{\gamma_{B}}\cdot I_{\gamma_{B}}^{FF}(0)\cdot S_{\phi_{\gamma_{B}\text{,eff}}}(0)}{c_{\alpha }\cdot I_{\alpha }^{FF}(0)+c_{\gamma_{B}}\cdot I_{\gamma_{B}}^{FF}(0)}.$$ (8)

#### 2. Local collective diffusion from neutron spin echo spectroscopy

For cases where q≈2π/d such as for neutron spin echo measurements, the above approach is not possible anymore, as *H*(*q*) is dependent on the structure and at the same time on the total volume fraction. We thus use the weighted average of the measured *q*-dependent collective diffusion coefficients $D_{\alpha }(q)$ and $D_{\beta }(q)$,

$$D(q)=\frac{I_{\alpha }(q)D_{\alpha }(q)+I_{\gamma_{B}}(q)D_{\gamma_{B}}(q)}{I_{\alpha }(q)+I_{\gamma_{B}}(q)},$$ (9)

where $I_{\alpha }(q)=c_{\alpha }I_{\alpha }^{FF}(q)S_{\phi_{\alpha ,\text{eff}}}(q)$ and $I_{\gamma_{B}}(q)=c_{\gamma_{B}}I_{\gamma_{B}}^{FF}(q)S_{\phi_{\gamma_{B},\text{eff}}}(q)$. $D_{\alpha }(q)$ and $D_{\gamma_{B}}(q)$ denote the measured diffusion coefficient at a volume fraction corresponding to the effective volume fraction $\phi_{\alpha ,\text{eff}}$ and $\phi_{\gamma_{B},\text{eff}}$, respectively. We remark that the measured diffusion coefficient thus do not contain the full hydrodynamic slowing down, so that a formal analogy to Eq. (5) is only preserved to a certain degree. While some shortcomings due to the neglected hydrodynamic slowing down at elevated volume fractions are to be expected, a thorough test how far these mixing rules can work is nevertheless of general interest.

### C. Viscosity

For viscosity measurements, we apply a simple Arrhenius relation for the relative viscosity $\eta_{r}$ of mixtures^57,58^ to theoretically predict $\eta_{r}(\phi_{\alpha \gamma_{B}})$ of the binary mixture from the relative viscosities of the individual protein solutions at their effective volume fraction $\eta_{r}(\phi_{\alpha ,\text{eff}})$ and $\eta_{r}(\phi_{\gamma_{\beta },\text{eff}})$,

$$\eta_{r}(\phi_{\alpha \gamma_{B}})=\eta_{r}(\phi_{\alpha ,\text{eff}})\cdot \eta_{r}(\phi_{\gamma_{B},\text{eff}}).$$ (10)

Here, we assume that a particular protein species *i* diffuses in a viscous medium formed by the other protein species *j* and the solvent. The viscosity of this effective solvent is given by that of the solution of the protein *j* at its effective volume fraction $\phi_{j,\text{eff}}$, i.e., by $\eta (\phi_{j,\text{eff}})$. The contribution of protein *i* to the total viscosity is then described by the corresponding relative viscosity of a solution of protein *i* at a volume fraction $\phi_{i,\text{eff}}$, i.e., by $\eta (\phi_{i,j})/\eta (\phi_{j,\text{eff}})=\eta_{r}(\phi_{i,\text{eff}})$. The individual values of $\eta_{r}(\phi_{\alpha ,\text{eff}})$ and $\eta_{r}(\phi_{\gamma_{B},\text{eff}})$ were obtained from the measured concentration dependence of $\eta_{r}(\phi_{\alpha })$ and $\eta_{r}(\phi_{\gamma_{B}})$ using a parameterization as described in supplementary material.

We stress that the simplicity of the mixing rules presented should not be seen as a serious drawback, but rather as a first test of how far simple concepts could be used to describe and predict the behavior of complex mixtures of different proteins under highly crowded conditions as often found in the cytosol of living cells or in blood serum. Section III presents different experimental quantities over a large range of concentrations, which are then compared with the predictions from these mixing rules. Importantly, these experimental quantities also encompass macro-, meso- as well as microscopic properties, and we will discuss the important consequences of these different length scales in the applicability of the mixing rules.

## III. RESULTS AND DISCUSSION

### A. Phase diagram

**FIG. 1.**
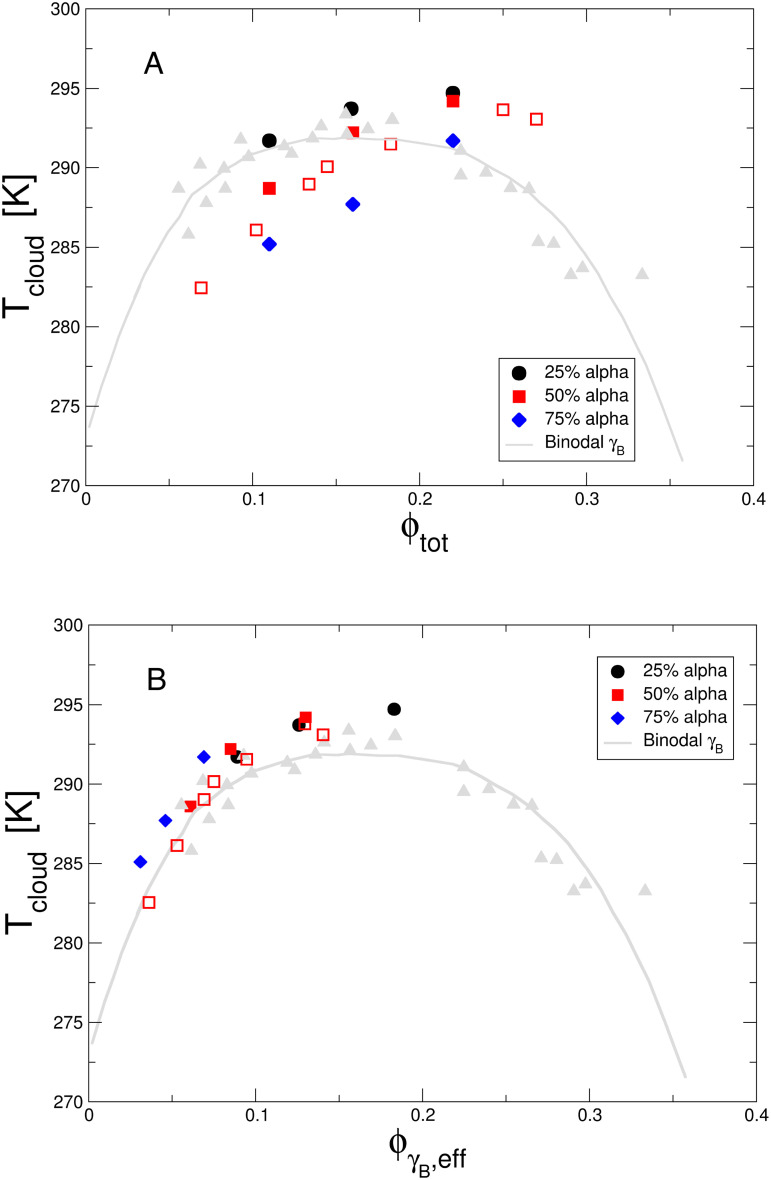
(a) Phase diagram for three $\alpha \gamma_{B}$ binary mixtures with volume fraction ratios of $\phi_{\alpha }:\phi_{\gamma_{B}}=3:1$ (blue diamonds), 1:1 (red squares), and 1:3 (black circles) in D_2_O phosphate buffer at pH = 7.1. Shown are the cloud point temperatures *T_cloud_* as a function of the total volume fraction $\phi_{\alpha \gamma B}$. Also shown is the binodal for pure *γ_B_* (gray triangles and gray solid line) (data for pure *γ_B_* crystallin taken from Ref. 25). Data for $\phi_{\alpha }:\phi_{\gamma_{B}}=1:1$ are from two different protein preparation batches (solid red squares batch 1, open red squares batch 2), and the data from batch 2 are shifted by 2.6 K in order to accommodate age and batch related shifts in the critical temperature of *γ_B_* crystallin. (b) Same data shown as a function of $\phi_{\gamma_{B},\text{eff}}$.

We first test our approach with a macroscopic property and investigate the location of the coexistence curve for liquid–liquid phase separation in $\alpha -\gamma_{B}$ protein mixtures. LLPS in the eye lens has been investigated intensively for its link to cataract formation and found to be directly related to $\gamma_{B}-\gamma_{B}$ protein–protein interactions.^6,8,46^ The results from our characterization of the location of the binodal for LLPS in these mixtures as determined from cloud point measurements for three different $\alpha :\gamma_{B}$ volume ratios (3:1, 1:1, and 1:3) are shown in Fig. 1(a).

When compared to the binodal in pure solutions of *γ_B_* crystallin from an earlier study^25^ (gray triangles), we see that an increasing amount of *α* crystallin has a significant effect, shifting the binodal to higher overall protein concentrations. It is interesting to look at this in view of the evolutionary pressure on the eye lens to maintain a cytosol composition that allows it to achieve high transparency at the very high protein concentrations required to generate the necessary index of refraction profile across the lens.^6,59^ As we will discuss below, the different crystallins allow indeed for a densely packed cell interior that optimizes index of refraction, transparency as well as viscosity.

If our reasoning in Sec. II is correct, we would expect to find the location of the binodal primarily determined by the relative volume fraction of *γ_B_* corrected by the reduced available volume caused by the *α* crystallins present, i.e., by $\phi_{\gamma_{B},\text{eff}}$ calculated using Eq. (2). This is demonstrated in Fig. 1(b), and we see that indeed all data points collapse around the binodal for the pure *γ_B_* system when using $\phi_{\gamma_{B},\text{eff}}$ instead of $\phi_{\alpha \gamma B}$.

The use of such a simple mixing rule based on the picture of a protein embedded in an effective solvent, where the additional other protein species present only result in a reduced available sample volume and thus an effectively increased volume fraction, is of course thermodynamically incorrect. We remark that a complete modeling of the phase diagram of $\alpha \gamma_{B}$ binary mixtures has in fact been performed previously.^29,51^ This work was also extended to a complete description of the resulting light scattering intensity based on a model of sticky spheres, allowing for a detailed investigation of the effects of protein–protein interactions ( $\alpha -\alpha ,\;\alpha -\gamma_{B}$ and $\gamma_{B}-\gamma_{B}$) on the intensity *I*(*q*) at *q* = 0.^60^ However, the resulting multidimensional phase diagrams become far too complex to be useful in a simple framework aiming at a qualitative understanding of the stability, the effect of temperature and composition, and the existence and location of an arrest line in crystallin mixtures with compositions similar to those in eye lens cells. Moreover, these calculations are difficult to extend to dynamic quantities such as the relative viscosity or the collective diffusion coefficient. We therefore aim to develop and test simple models to describe phenomenological observations in a semi-quantitative way, amenable for numerical calculations of thermodynamic, structural and dynamic properties that could not be achieved otherwise and would require extensive computer simulations instead.

### B. Viscosity

Next, we extend our approach to a macroscopic dynamic quantity and investigate the relative zero-shear viscosity $\eta_{r}$ of $\alpha \gamma_{B}$ binary mixtures. We employ measurements using both DLS-based and MPT-based microrheology, as described in the Materials and Methods section. To ensure that experiments are made on mixtures in the one-phase region of the phase diagram, all measurements are performed at temperatures well above *T_cloud_*.

**FIG. 2.**
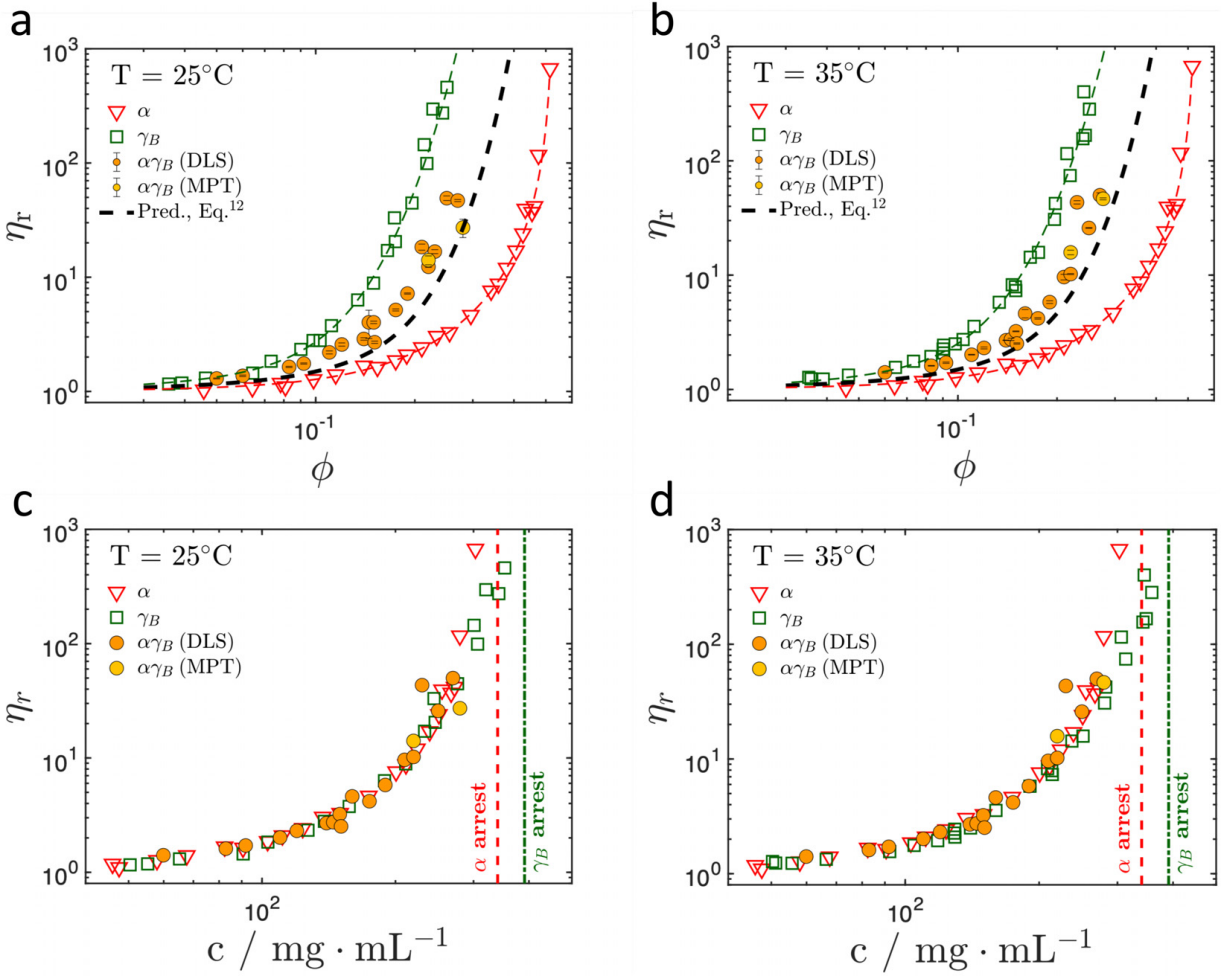
(a) and (b). *η_r_* vs volume fraction ϕ for the pure crystallins (taken from Refs. 49 and 61) and the $\alpha \gamma_{B}$ binary mixture in D_2_O, taken at T= 25 and 35 °C. The colored dashed lines represent the parameterized results for the pure crystallins (see supplementary material for details). The black dashed line shows the results of the theoretical prediction for the binary mixtures [Eq. (10)]. (c) and (d). Relative viscosity *η_r_* vs protein total concentration for pure *α* and *γ_B_* crystallins and $\alpha \gamma_{B}$ binary mixture at T= 25 and 35 °C, respectively. Also shown are the locations of the arrest lines for pure *α* and *γ_B_* crystallin solutions.

Figures 2(a) and 2(b) report $\eta_{r}$ for the pure *α* crystallin (red triangles) and *γ_B_* crystallin (green squares) solutions as well as for the 1:1 binary mixture as a function of volume fraction ϕ at T= 25 and 35 °C. The results for the $\alpha \gamma_{B}$ binary mixture are shown as orange (DLS-based microrheology) and yellow (MPT-based microrheology) circles. Representative $g_{2}(q,\tilde{t})-1$ functions from DLS-based microrheology, as well as mean-squared displacements and Van Hove functions from multiple particle tracking (MPT)-based microrheology are reported in Fig. S1 in supplementary material.

The volume fraction dependence of $\eta_{r}$ of the mixture is well reproduced by the mixing rule in Eq. (10) [Figs. 2(a) and 2(b)]. (see supplementary material for a detailed description). The combination of the $\eta_{r}$ of *α* and *γ_B_* crystallins taken at their effective volume fractions $\phi_{\gamma_{B},\text{eff}}$ and $\phi_{\alpha ,\text{eff}}$ indeed appears to provide a good prediction of the binary mixture. This is quite remarkable given the very different mechanisms behind dynamical arrest for the two different lens proteins. While *α* crystallin exhibits a hard sphere-like behavior with an arrest transition at around $\phi_{g,\alpha }\approx 0.58$,^24^ *γ_B_* crystallin has been found to show a behavior described by a model of particles with a weak short range attraction combined with additional attractive patches. This results in a dynamical arrest transition at significantly lower volume fractions of $\phi_{g,\gamma_{B}}\approx 0.3-0.35$, presumably caused by the presence of transient clusters.^19,25^ This good agreement suggests that on macroscopic length scales probed by the zero shear viscosity, and given strongly differing viscosities, viscoelastic relaxations arising from protein–protein interactions can be decoupled for the two proteins. At the effective volume fractions $\phi_{\alpha ,\text{eff}}\ll \phi_{g,\alpha }$ measured for our binary mixtures, *α* crystallin solutions have rather low viscosities and relax quickly, allowing for a treatment of the *α* crystallin as an effective solvent for *γ_B_* crystallin, which shows significantly higher viscosity and slower relaxation times at these effective volume fractions due to the formation of transient clusters.

Due to the attractive interactions between *γ_B_* crystallins, their solutions undergo LLPS at lower temperatures as demonstrated in Fig. 1. This also has consequences for scattering experiments, as there will be a significant contribution from critical scattering, resulting in a strongly enhanced intensity in SLS and a slowing down of the measured correlation function obtained in DLS as the temperature approaches *T*_cloud_.^25^ While these additional contributions from critical opalescence and critical slowing down make microrheology measurements using DLS-based tracer microrheology much more difficult and induce additional errors (see supplementary material), this can be overcome by using MPT-based microrheology instead. However, while temperature has a strong effect on the phase behavior and the results from scattering experiments, the relative viscosity shows no measurable temperature dependence. This indicates that there are no or only weak critical contributions to the $\eta_{r}$ of binary *α* - *γ_B_* mixtures.

An interesting feature is observed when plotting and comparing relative viscosity data for pure crystallins and the binary mixture as a function of mass concentration *c* in mg/mL. As shown in Figs. 2(c) and 2(d), $\eta_{r}$ from the different systems collapse onto a master curve that extends to rather high concentrations. This coincidence suggests that for these specific proteins, $\eta_{r}$ is determined by the actual weight concentration for low to intermediate protein concentrations, independent of the ratio between the pure components. It is important to point out that this $\eta_{r}$ master curve holds up to concentrations *c* of around 300 mg/mL. For higher *c*, $\eta_{r}$ diverges at the arrest value *c_g_* of the particular protein species or mixture, which is different for the two proteins. In particular, for *α* crystallin the arrest transition occurs at ≈ 341 mg /mL [red dashed lines in Figs. 2(c) and 2(d)], while dynamical arrest for *γ_B_* crystallin occurs at ≈ 395 mg/mL [green dashed lines in Figs. 2(c) and 2(d)]. However, it is important to point out that we do not expect to find such a behavior for arbitrary proteins and consider this an effect of the very different voluminosities of *α* and *γ_B_* crystallin that compensate for the different underlying arrest mechanisms. Instead, we expect that a mixing rule such as given in Eq. (10), where effective volume fractions are considered, should have a much wider applicability.

### C. Collective gradient diffusion

Next, we move on to mesoscopic length scales of many protein diameters and investigate collective gradient diffusion of $\alpha \gamma_{B}$ binary mixtures using dynamic light scattering. The protein solution is no longer considered to behave as an effective homogeneous liquid, but as a concentration field, in which concentration fluctuations or inhomogeneous density distributions relax with a rate expressed through the gradient diffusion coefficient D=D(ϕ,q→0).

**FIG. 3.**
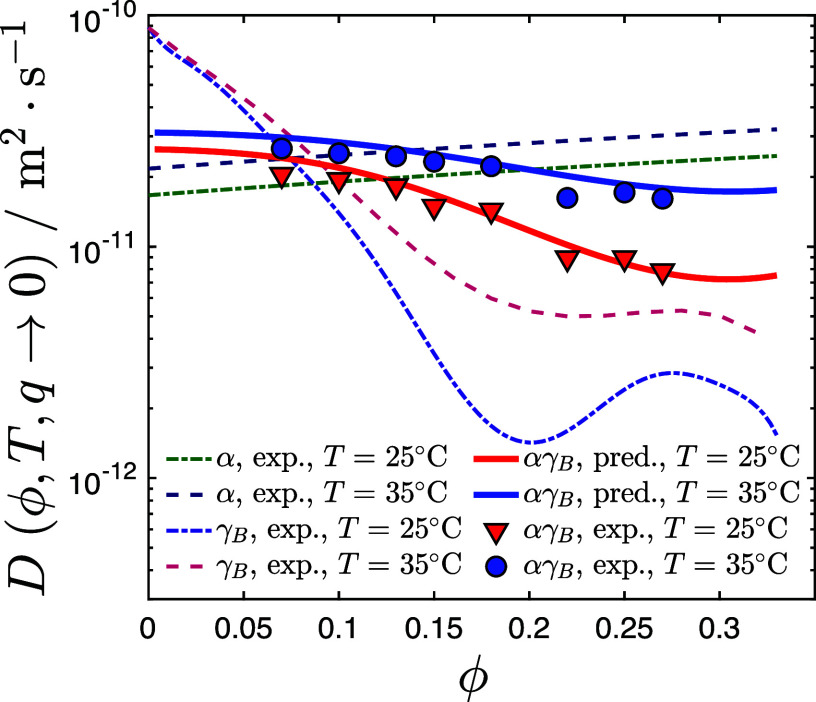
Gradient diffusion coefficients of 1:1 $\alpha \gamma_{B}$ mixtures [ $D_{\alpha \gamma_{B}}(\phi ,q\to 0)$] and pure components [ $D_{\alpha }(\phi ,q\to 0),\;D_{\gamma_{B}}(\phi ,q\to 0)$)] in the q→ 0 limit at two temperatures and as a function of volume fraction. Lines: theoretical diffusion coefficients for binary mixtures calculated as described in the text (solid lines), and interpolated values from DLS experiments with the pure crystallins (dashed lines). Solid symbols: collective diffusion coefficients obtained via DLS measurements at *T* = 25 °C (red triangles) and 35 °C (blue circles).

Figure 3 presents the measured diffusion coefficients $D_{\alpha \gamma_{B}}(\phi_{\alpha \gamma_{B}})$ for the 1:1 mixture at two temperatures T=25 and 35 °C for a series of volume fractions $\phi_{\alpha \gamma_{B}}$. For both temperatures, we observe a decrease in $D(\phi_{\alpha \gamma_{B}})$ with increasing volume fraction. Also shown is the concentration dependence of the pure components $D_{\alpha }(\phi_{\alpha })$ and $D_{\gamma_{B}}(\phi_{\gamma_{B}})$, respectively.^24,25^

While *α* crystallin solutions exhibit a concentration dependence of D(ϕ) that is typical for hard spheres with an almost linear increase in D(ϕ) with increasing volume fraction,^24^ the ϕ-dependence of D(ϕ) is much more complex for *γ_B_* crystallin. For this protein, we find a non-monotonic ϕ-dependence due to the combination of contributions from a strongly *T*-dependent critical slowing down in the vicinity of the critical point and a regular non-critical contribution leading to dynamical arrest at ϕ≈0.35.^25^ For the 1:1 $\alpha \gamma_{B}$ mixture, we observe a behavior that is roughly intermediate between the two individual protein solutions.

We now attempt to use Eq. (5) together with the simple mixing rules given in Eqs. (6)–(8) in order to calculate the theoretical predictions for the mixed system. To determine *D*_0_ for the simple mixing rule in Eq. (5), we use $D_{0,\alpha }=$ 2.2 × 10^−11^ and $D_{0,\gamma_{B}}=$ 8.74 × 10^−11^ m^2^ s^−1^ at T=20 °C, which we then adapt for the correct solvent viscosities at the temperatures measured (see supplementary material for details).^24,25^ For *S*(0) in the case of *α* crystallin, we employ the Carnahan-Starling equation for monodisperse hard spheres $S_{\text{CS}}(0)=\left[{\left(1-\phi \right)}^{4}\right]/\left[{\left(1+2\cdot \phi \right)}^{2}+\phi^{3}\cdot (\phi -4)\right]$.^24^ $S_{\gamma_{B}}(0)$ for each temperature are taken from Ref. 25 and parameterized with a simple polynomial function.

The agreement between the measured concentration dependence of the gradient diffusion coefficient $D(\phi_{\alpha \gamma_{B}})$ and the predictions from Eq. (5) together with the simple mixing rules given in Eqs. (6)–(8) is quite remarkable, especially when considering that the individual proteins exhibit such a vastly different behavior (Fig. 3). We believe that the hybrid approach used here is essential for this, where we calculate the thermodynamic driving force for the decay of concentration fluctuations given by the osmotic compressibility *S*(0) based on the particular α−α and $\gamma_{B}-\gamma_{B}$ interactions using the effective volume fractions of both protein components, while we estimate the contributions from hydrodynamic interactions from the overall volume fraction. This allows us to also incorporate contributions from critical fluctuations for proteins that undergo LLPS, while the frictional resistance that the different proteins experience should primarily depend on the total volume fraction of proteins in solution.

### D. Solution structure

After having successfully reproduced macro- and mesoscopic thermodynamic and dynamic properties, we next investigate whether the proposed mixing rules can also describe structural and dynamic solution properties on more local or molecular length scales. We first investigate the solution structure on length scales from a fraction of the protein diameter up to a few protein molecules as obtained from SAXS measurements.

We first verify that Eq. (4) correctly reproduces the measured SAXS intensity *I*(*q*) for protein mixtures in the absence of contributions from protein–protein interactions, i.e., in the limit of infinite dilution where $S_{\phi_{\alpha ,\text{eff}}}(q)=S_{\phi_{\gamma_{B},\text{eff}}}(q)=1$. Under these conditions, we measure an effective form factor $I_{\alpha \gamma_{B}}^{\text{FF}}(q)$ that corresponds to an intensity-weighted average of the two individual form factors of *α* and *γ_B_* crystallin given by

$$I_{\alpha \gamma_{B}}^{\text{FF}}(q)=\underset{c_{\alpha \gamma_{B}}\to 0}{\text{lim}}\frac{I_{\alpha \gamma_{B}}(q)}{c_{\alpha \gamma_{B}}}=\underset{c_{\alpha \gamma_{B}}\to 0}{\text{lim}}\frac{c_{\alpha }\cdot I_{\alpha }^{\text{FF}}(q)+c_{\gamma_{B}}\cdot I_{\gamma_{B}}^{\text{FF}}(q)}{c_{\alpha }+c_{\gamma_{B}}}.$$ (11)

**FIG. 4.**
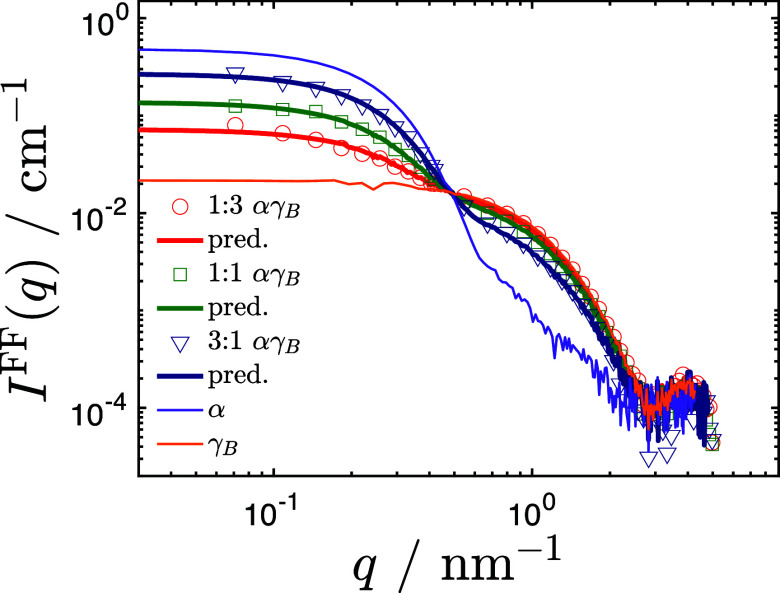
Scattering intensity from SAXS for $\alpha \gamma_{B}$ binary mixtures at different *α* (or *γ_B_*) ratios and a total ϕ= 0.003. Red, green, and blue open symbols identify the 1:3, 1:1, and 3:1 ratios, respectively. Form factors for pure crystallins are indicated as purple lines for *α* crystallin and orange lines for *γ_B_* crystallin. Colored solid lines are the resultant fits from Eq. (11).

Figure 4 shows the concentration-normalized form factor $I_{\alpha \gamma_{B}}^{\text{FF}}(q)$ from SAXS for dilute solutions of $\alpha \gamma_{B}$ binary mixtures at different *α*:*γ_B_* mixing ratios, i.e., 1:3 (red circles), 1:1 (green squares), and 3:1 (blue triangles). At this dilute volume fraction of $\phi_{\alpha \gamma_{B}}=0.003$, effects from protein–protein interactions can be neglected. This is indeed demonstrated in Fig. 4, where we see that the scattering from the mixed solutions is quantitatively reproduced by a linear combination (color-matched lines) of the individual form factors for the pure *α* (purple line) and *γ_B_* (orange line) crystallins also shown in Fig. 4. This also clearly indicates the absence of any self-association under these conditions.

**FIG. 5.**
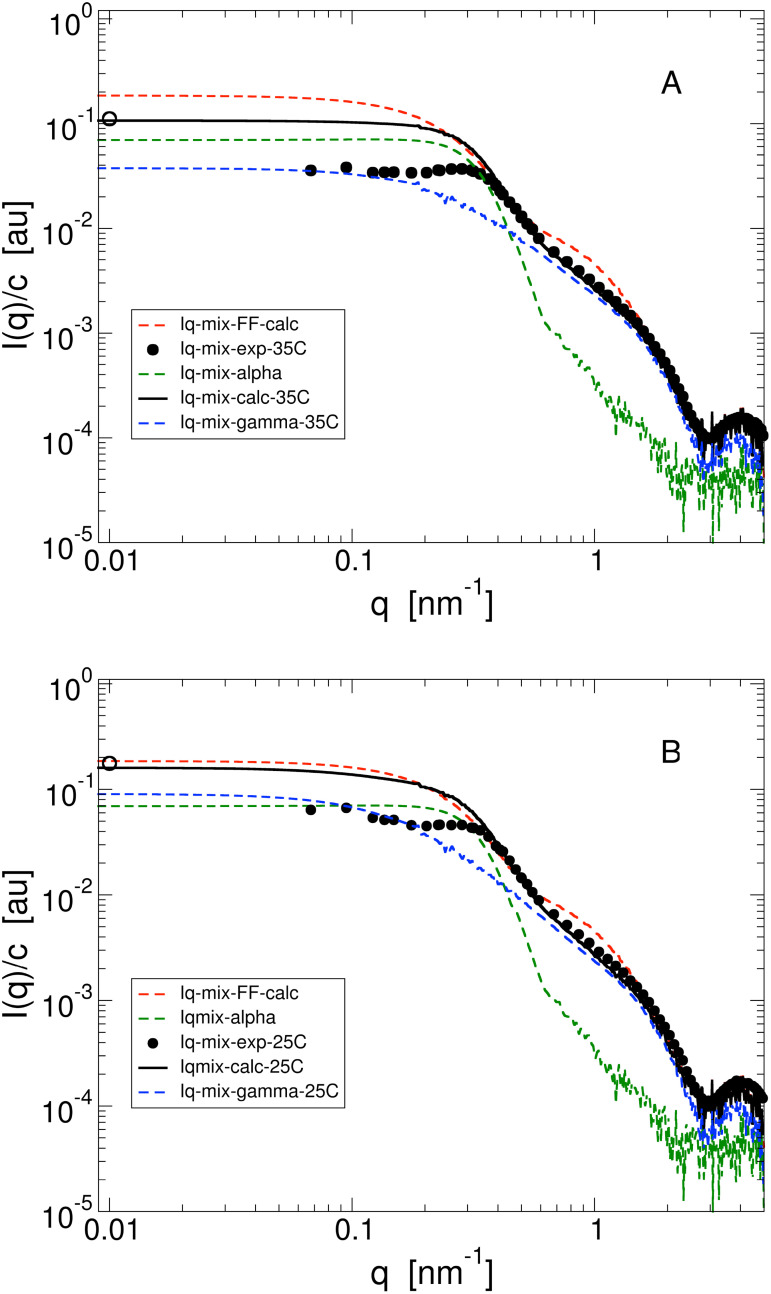
Concentration-normalized scattering intensity $I_{\alpha \gamma_{B}}(q)/c_{\alpha \gamma_{B}}$ from SAXS (solid black circles) and SLS (open black circle) for $\alpha :\gamma_{B}=1:1$ binary mixtures at a total volume fraction ϕ=0.22 for T=35 (a) and T=25 °C (b). Also shown are the predicted q− dependence from Eq. (4) (solid black line), the case for no interactions given by Eq. (11) (dashed red line), and the contributions from *α* (dashed green line) and *γ_B_* (dashed blue line) given by $I_{\alpha }(q)/c_{\alpha \gamma_{B}}=c_{\alpha }\cdot I_{\alpha }^{\text{FF}}(q)\cdot S_{\phi_{\alpha ,\text{eff}}}(q)/c_{\alpha \gamma_{B}}$ and $I_{\gamma_{B}}(q)/c_{\alpha \gamma_{B}}=c_{\gamma_{B}}\cdot I_{\gamma_{B}}^{\text{FF}}(q)\cdot S_{\phi_{\gamma_{B},\text{eff}}}(q)/c_{\alpha \gamma_{B}}$.

We next characterize the concentration and temperature dependence of the solution structure for 1:1 mixtures using SAXS. Figures 5(a) and 5(b) show the experimental SAXS curves and their theoretical predictions from Eq. (4) for $\alpha :\gamma_{B}=1:1$ binary mixtures at $\phi_{\alpha \gamma_{B}}=0.22$ and two temperatures T=25 (a) and T=35 °C (b), respectively.

The theoretical predictions are based on the scattering intensities measured for the pure *α* and *γ_B_* crystallin solutions using the mixing rule in Eq. (4) and their effective volume fractions $\phi_{\alpha ,\text{eff}}$ and $\phi_{\gamma_{B},\text{eff}}$ [Eqs. (1) and (2)], respectively. This requires theoretical expressions for the structure factors of the pure components $S_{\phi_{\alpha \text{,eff}}}(q)$ and $S_{\phi_{\gamma_{B},\text{eff}}}(q)$.

For the structure factor of *α* crystallin, we use the Percus–Yevick approximation for polydisperse hard spheres, where the particle size distribution is described with a Schulz distribution with an average particle diameter of 15 nm and a polydispersity of 20%, which has been shown to describe $S_{\alpha }(q)$ with very good accuracy on the entire accessible volume fraction range up to the arrest transition at $\phi_{g,\alpha }\approx 0.58$.^24^ For pure *α* crystallin solutions, we moreover expect no measurable temperature dependence of $S_{\phi_{\alpha }}(q)$ for the temperatures investigated.

For *γ_B_* crystallin solutions, the situation is more complex, as the additional attraction between proteins results in a very strong and non-monotonic temperature and concentration dependence of $S_{\phi_{\gamma_{B}}}(q)$ in the vicinity of the critical point. As previously reported in the literature,^25^ the structure factor of *γ_B_* crystallin is well described by a combination of two contributions from critical and non-critical scattering given by

$$S_{\gamma_{B}}(q,T,\phi )=S_{\text{crit}}(q,T,\phi )+S_{\text{non}\;\text{crit}}(q,\phi ),$$ (12)

at $q≲\frac{2\pi }{d}$, with the protein diameter $d_{\gamma_{B}}\approx 4$ nm. $S_{\text{crit}}$ describes the contributions from critical concentration fluctuations and thus strongly depends on temperature and concentration, primarily given by the distance of the particular state point from the critical point or spinodal line. The second term $S_{\text{non}\;\text{crit}}$ represents the *q*-dependent background, related to the athermal excluded volume interactions.

We calculate $S_{\gamma_{B}}(q,T,\phi )$ with the following expression:

$$S_{\gamma_{B}}(q,T,\phi )=\left[\frac{S_{\text{crit}}(0)}{1+q^{2}\xi^{2}}\right]+\left[\frac{1}{1+\frac{1-S_{\text{non}\;\text{crit}}(0)}{S_{\text{non}\;\text{crit}}(0)}\cdot P_{\gamma_{B}}(q)}\right].$$ (13)

The first term is a simple Ornstein–Zernike relation to describe the contributions from critical scattering in Eq. (13), where $S_{\text{crit}}(0)$ is given by the osmotic compressibility and *ξ* is the static correlation length.^25^ For the non-critical contribution, we consider exclude volume interactions between the ellipsoid-shaped *γ_B_* crystallins only, which we describe using a so-called random-phase approximation, where $P_{\gamma_{B}}(q)$ is the form factor of *γ_B_*, normalized to I(q,0)=1. The actual values used are taken from a detailed investigation of the structural and dynamic properties of *γ_B_*-crystallin described in Ref. 25. More details about the procedure and an example of the different contributions to the scattering intensity from a *γ_B_* crystallin solution can be found in supplementary material.

The comparison between the experimental data and the calculated curves shown in Fig. 5 reveals good agreement for higher *q*-values at q≳0.5 nm^−1^, and also the low-*q* limit $I(0)/c_{\alpha \gamma_{B}}$ obtained from SLS is quite well reproduced at both temperatures. However, at intermediate *q*-values around 0.07 ${\text{nm}}^{-1}≲q≲0.5$ nm^−1^ we observe systematic differences for both temperatures. There appears to be a more prominent local structure on length scales of the *α*-crystallin diameter than predicted by our mixing rule model. In order to better understand the origins of this discrepancy, it is quite instructive to look at the individual contributions to the scattering intensity arising from the two proteins also shown in Fig. 5. The high-*q* part of $I_{\alpha \gamma_{B}}(q)/c_{\alpha \gamma_{B}}$ obtained from SAXS is dominated by the scattering from *γ_B_*-crystallin, while the scattering from *α*-crystallin dominates for 0.1 ${\text{nm}}^{-1}≲q≲0.4$ nm^−1^, i.e., close to the nearest neighbor peak of *α*-crystallin solutions at comparable protein volume fractions. Finally, at low-*q* values it depends on temperature whether the scattering for *α*-crystallin or *γ_B_*-crystallin dominates due to the strong dependence of $S_{\text{crit}}(q,T,\phi )$ in Eq. (12).

**FIG. 6.**
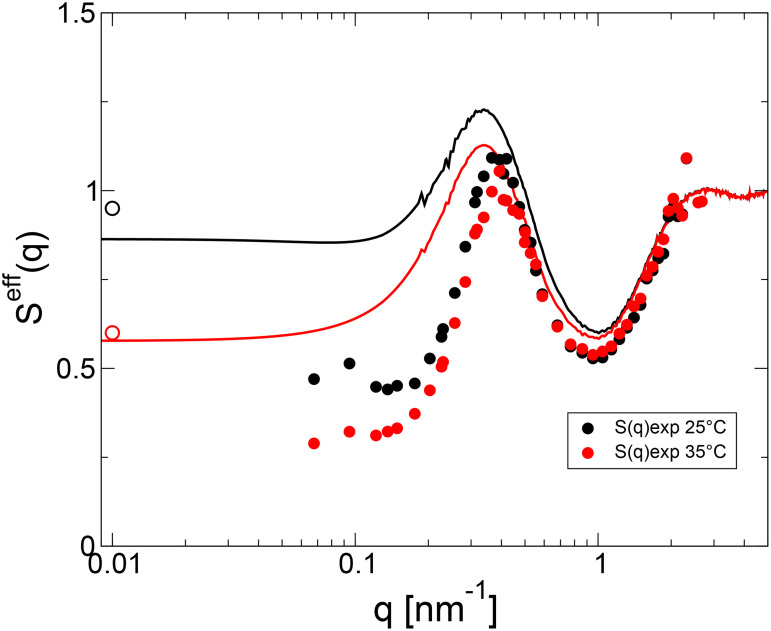
The measured effective structure factors $S^{\text{eff}}(q)=[I_{\alpha \gamma_{B}}(q)/c_{\alpha \gamma_{B}}]/I_{\alpha \gamma_{B}}^{FF}(q)$ together with the predictions based on Eq. (8) where we use the full *q*-dependence instead of only the *q* = 0 limit. Open (SLS) and solid (SAXS) symbols are for the experimental results, solid lines for the predictions given by Eq. (8). T=35 °C: red color and T=25 °C black color.

In order to better understand the limits of our mixing rule approach, we also calculate the measured effective structure factor using

$$S^{\text{eff}}(q)=[I_{\alpha \gamma_{B}}(q)/c_{\alpha \gamma_{B}}]/I_{\alpha \gamma_{B}}^{FF}(q),$$ (14)

where $I_{\alpha \gamma_{B}}(q)$ corresponds to the measured scattering intensity of the mixture, $c_{\alpha \gamma_{B}}$ is the total weight concentrations and $I_{\alpha \gamma_{B}}^{FF}(q)$ is the measured concentration-normalized form factor of the mixture. The measured values of $S^{\text{eff}}(q)$ are shown in Fig. 6, together with the calculated values based on the mixing rule approach. Here we use

$$S^{\text{eff}}(q)=\frac{c_{\alpha }\cdot I_{\alpha }^{FF}(q)\cdot S_{\phi_{\alpha \text{,eff}}}(q)+c_{\gamma_{B}}\cdot I_{\gamma_{B}}^{FF}(q)\cdot S_{\phi_{\gamma_{B}\text{,eff}}}(q)}{c_{\alpha }\cdot I_{\alpha }^{FF}(q)+c_{\gamma_{B}}\cdot I_{\gamma_{B}}^{FF}(q)}.$$ (15)

to calculate $S^{\text{eff}}(q)$, where $I_{\alpha }^{FF}(q)$ and $I_{\gamma_{B}}^{FF}(q)$ are the measured form factors of the two proteins, while $S_{\phi_{\alpha \text{,eff}}}(q)$ and $S_{\phi_{\gamma_{B}\text{,eff}}}(q)$ are calculated as described above.

Figure 6 indeed shows that the local structure is well reproduced for q≳0.5 nm^−1^, where the scattered intensity from *α* crystallins has strongly decreased due to the form factor of the large protein, and where the data thus primarily describe the local structural correlations between *γ_B_* crystallins. Here, $S_{\phi_{\gamma_{B}\text{,eff}}}(q)$ is also almost independent of temperature, as these local correlations are dominated by excluded volume effects. At low *q*-values, $S^{\text{eff}}(q)$ exhibits a clear temperature dependence, reflecting the enhanced contribution form critical scattering from *γ_B_* with decreasing temperature. Moreover, on length scales comparable to the *α* crystallin diameter and larger, the structural correlations are much more pronounced than predicted. Furthermore, the additional low-*q* value from SLS indicates the existence of additional weak attractions that result in a significant upturn of $S_{\phi_{\gamma_{B}\text{,eff}}}(q)$ at low *q*. The *γ_B_* contributions from critical fluctuations and the resulting static correlation length at these temperatures are too small to cause this considerable correlation hole and subsequent strong upturn at low *q*. However, previous results from SANS experiments and computer simulations of $\alpha -\gamma_{B}$ mixtures have provided compelling evidence for the presence of mutual weak attractions between these two proteins, resulting effectively in a higher stability against phase separation when compared to a situation where the two protein species interact via excluded volume interactions only.^28,50^ Computer simulations that allow for the calculation of the different partial structure factors [ $S_{\alpha \alpha }(q),\;S_{\alpha \gamma_{B}}(q)$, and $S_{\gamma_{B}\gamma_{B}}(q)$ in Eq. (3)] indeed show that $S_{\alpha \alpha }(q)$ exhibits a behavior that is strongly reminiscent of weakly attractive hard spheres, where the *γ_B_* proteins act as temporary bonds between two *α* crystallins.^28^

**FIG. 7.**
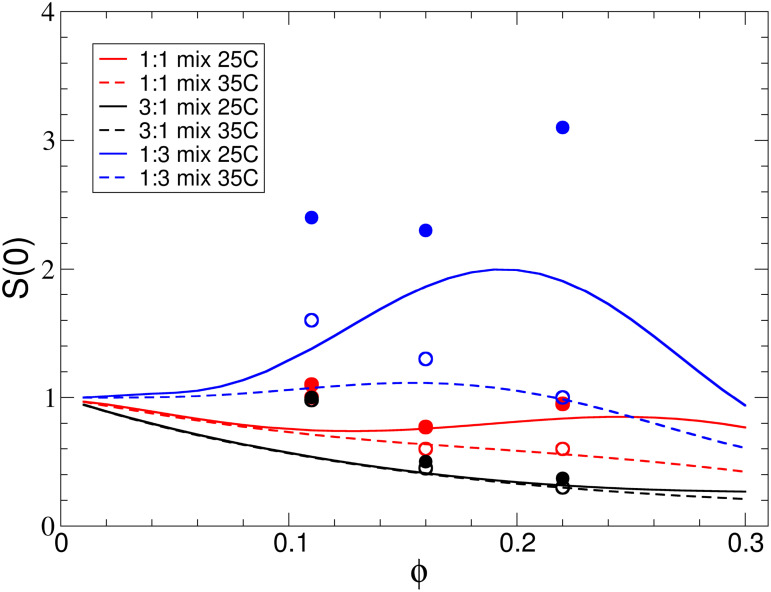
Osmotic compressibility *S*(0) from SLS for $\alpha \gamma_{B}$ binary mixtures at different *α*:*γ_B_* mixing ratios, total volume fractions ϕ= and two temperatures T=25 and T=35 °C, respectively. Measured data are given by the open and filled symbols, while the predicted values based upon the mixing rule given by Eq. (8) are shown as the solid and dashed lines. Black, red, and blue symbols and lines identify the *α*: $\gamma_{B}=3:1,\;1:1$, and 1:3 mixing ratios, respectively. Open symbols and dashed lines are for T=25 °C, and solid lines and filled symbols for T=35 °C.

It is quite intriguing that the mixing rule approach reproduces the osmotic compressibility of the mixture quite well, despite the fact that local correlations are not well described and strongly underestimated on length scales of the *α* crystallin diameter. We have characterized *S*(0) for different mixing ratios, temperatures and total protein volume fraction using SLS, and the results are summarized in Fig. 7. Here, we see that Eq. (8) indeed reproduces the measured compressibility quite well for the higher total volume fractions ϕ=0.16 and ϕ=0.22, but fails to do so at the lowest volume fraction ϕ=0.11. Moreover, the agreement is also less good for the mixing ratio *α*: $\gamma_{B}=1:3$ and the lower temperature T=25 °C, where the measured values are much higher than predicted by the mixing rule approach. At high concentrations and higher temperatures where critical fluctuations are not important, the compressibility is primarily determined by strong excluded volume effects between the different proteins, which is reasonably described by our approach. On the other hand, addition of a smaller amount of *α* crystallin to *γ_B_* crystallins enhances the criticality of *γ_B_* solutions in a non-monotonic way, with a maximum at a mixing ratio of 1:3. Under these conditions, the mixture phase separates at a higher temperature, and critical scattering is thus significantly enhanced already at T=25 °C.^28^ Moreover, the effects of an additional weak attraction on *S*(0) become much more visible at lower volume fractions, where *S*(0) shows also a non-monotonic behavior for attractive hard sphere systems with a maximum that depends on the nature and strength of the attraction.^62^ With *γ_B_* crystallins acting as temporary bonds between *α* crystallins, this can lead to enhanced scattering even far away from phase separation, and thus qualitatively explain the failure of the mixing rule calculations at lower total protein concentrations.

The analysis of the SAXS data has clearly demonstrated the limits of the simple mixing rules in describing the properties of protein mixtures at high concentrations, i.e., under crowded conditions. While they were able to reproduce the experimental data for the macroscopic and mesoscopic thermodynamic and dynamic properties almost quantitatively, they fail to describe the microscopic structural features for higher concentrations in the 1:1 mixture. This clearly indicates that we would need to explicitly consider the different partial structure factors, which would require us to properly treat also mutual interactions between *α* and *γ_B_* crystallins. This is also in line with earlier simulation work, which had pointed out that a weak mutual attraction between *α* and *γ_B_* crystallins resulted in an enhanced stability of the mixture, suppressing LLPS and increasing transparency of the solution. However, it is also interesting to note that these mixing rules are able to reproduce the osmotic compressibility quite well at high concentrations, i.e., under conditions existing in living cells, as long as the solution conditions are not close to a boundary for LLPS for some of its proteins.

### E. Collective diffusion on the local scale

As a final test, we explored collective diffusion on the microscopic length scale of individual molecules using neutron spin echo spectroscopy, i.e., the relaxation of density correlations such as cage formation in concentrated protein solutions.

We collected intermediate scattering functions *I*(*q*, *t*) for mixed solutions of *α* and *γ_B_* crystallin at volume fractions of 0.11 and 0.22. We used several *q* values covering both the correlation hole below 0.1 nm^−1^ and the nearest-neighbor scale of *γ_B_* crystallin beyond 2 nm^−1^. From the rate constant Γ(q) of the initial decay of the measured correlation functions *I*(*q*, *t*), we obtained *q*-dependent diffusion coefficients $D(q)=\Gamma /q^{2}$ (cf. supplementary material for details).

**FIG. 8.**
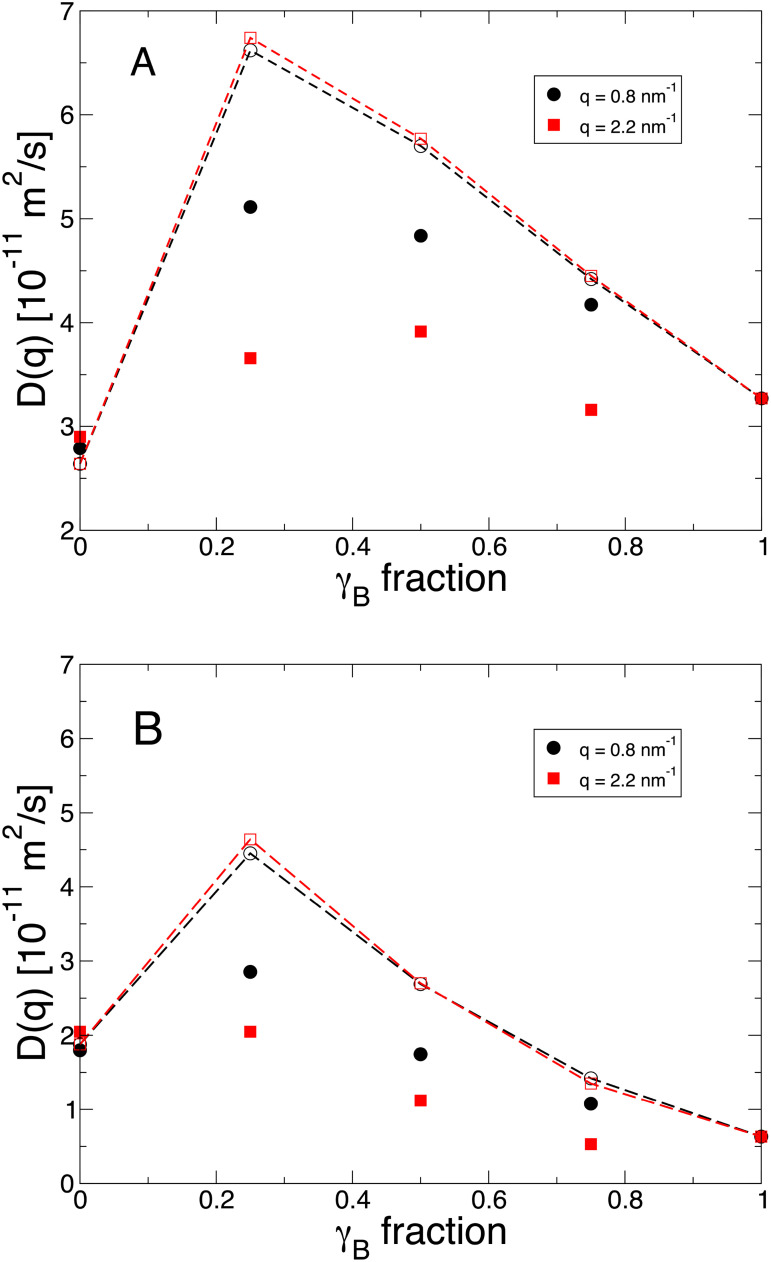
Diffusion coefficients *D*(*q*) from neutron spin echo spectroscopy in mixtures of *α* and *γ* crystallin as a function of total volume fraction and composition for two *q*-values (black symbols: *q* = 0.8 nm^−1^; red symbols: *q* = 2.2 nm^−1^. Solid symbols are measured and open symbols calculated values. The dashed lines connecting the calculated values are drawn as guides to the eye only. All data were measured at 35 °C. (a) ϕ=0.11 and (b): ϕ=0.22.

Figure 8 displays the results as a function of the fraction of *γ_B_* crystallin (symbols). We observe clear non-monotonous trends of the diffusion coefficients with increasing *γ* fraction. In all cases, the 3:1 $\alpha :\gamma_{B}$ mixtures relax faster than pure *α* crystallin. From this maximum value, *D*(*q*) decays to the value for pure *γ_B_* crystallin. While the signatures at different *q* values differ mainly by an offset, the observed trend is significantly varied between the two explored volume fractions.

It has previously been reported that for proteins such as *γ_B_* crystallin experiencing patchy attractions, collective diffusion at length scales of the nearest neighbor distance can be dramatically altered when compared to the predictions for simple colloid models based on an isotropic potential due to the formation of transient clusters.^19^ While collective diffusion on mesoscopic length scales measured by DLS could be well described by a simple mixing rule given by Eq. (5), we can no longer use such an approach. Instead we calculate a weighted average of the diffusion coefficients of the pure systems $D_{\alpha }(q)$ and $D_{\gamma_{B}}(q)$ at their respective effective volume fractions $\phi_{\alpha ,\text{eff}}$ and $\phi_{\gamma_{B},\text{eff}}$ as given by Eq. (9). The thus predicted values of *D*(*q*) indeed show a qualitatively similar behavior as observed in the NSE experiments.

This particular dependence on composition is a result of the combination of the very different scattering intensities and diffusion coefficients for the two proteins. At both *q*-values, the scattering intensity from *γ_B_* crystallin is much higher than that of *α* crystallin, resulting in ratios of $I_{\alpha }(q)/I_{\gamma_{B}}(q)$ as summarized in Table II. At the same time, the collective diffusion coefficient $D_{\gamma_{B}}(q)$ exhibits a much stronger concentration dependence than $D_{\alpha }(q)$ (see supplementary material), resulting in a non-monotonic dependence of the intensity weighted *D*(*q*) as a function of the mixing ratio.

**TABLE II.** Ratio of the total scattering contribution from *α* crystallin to that of *γ_B_* crystallin, $I_{\alpha }(q)/I_{\gamma_{B}}(q)$, calculated using $I_{\alpha }(q)=c(\alpha )I_{\alpha }^{FF}(q)S_{\phi_{\alpha ,\text{eff}}}(q)$ and $I_{\gamma_{B}}(q)=c(\gamma_{B})I_{\gamma_{B}}^{FF}(q)S_{\phi_{\gamma_{B},\text{eff}}}(q)$, for different mixing ratios, at two total volume fractions and *q*-values.

$\phi_{\alpha }:\phi_{\gamma_{B}}$	$I_{\alpha }(q)/I_{\gamma_{B}}(q)$	$I_{\alpha }(q)/I_{\gamma_{B}}(q)$	$I_{\alpha }(q)/I_{\gamma_{B}}(q)$	$I_{\alpha }(q)/I_{\gamma_{B}}(q)$
	ϕ=0.11	ϕ=0.11	ϕ=0.22	ϕ=0.22
	*q* = 0.8 nm^−1^	*q* = 2.2 nm^−1^	*q* = 0.8 nm^−1^	*q* = 2.2 nm^−1^
3:1	0.45	0.43	0.52	0.43
1:1	0.16	0.14	0.22	0.14
1:3	0.06	0.05	0.09	0.05

However, while the qualitative trend is correctly reproduced, the actual values calculated are systematically overestimating the measured *D*(*q*). This is particularly pronounced for the 3:1 mixture. Moreover, the measured *q*-dependence of *D*(*q*) is much more pronounced than predicted by Eq. (9), with significantly lower values of *D*(*q*) at *q* = 2.2 nm^−1^ than at *q* = 0.08 nm^−1^.

The diffusion coefficients measured by NSE represent short-time collective relaxations on the nearest neighbor distance and are governed by the local neighbor structure as well as by hydrodynamic interactions. The effective static structure factor of the mixture $S^{\text{eff}}(q)$ is quite well reproduced by our mixing rule approach at these *q*-values (see Fig. 6), indicating that the local structure on these rather short length scales of order the size of *γ_B_* crystallin are not too different from those underlying the mixing rules used, i.e., assuming that they are determined primarily by the *γ_B_*–*γ_B_* and *α*–*α* interactions at their corresponding effective volume fractions. On the other hand, in our approach we consider effective volume fractions only, i.e., we treat the other protein species as immobile obstacles that reduce the available free volume but do not contribute to hydrodynamic interactions. While this explains some of the overestimation of *D*(*q*) by our model, it can of course not explain the significant *q*-dependence in the measured data.

For a puristic theoretical understanding, one would need all pair-wise partial structure factors, which are not available for the given experimental systems. While a complete understanding of these signatures would thus require comprehensive simulations or complicated parametrizations of complex colloid theory that also takes into account possible patchy interactions between different proteins, this deviation from the simplistic mixing rule implies that mutual interactions between the two proteins actually strongly affect the dynamics on local length scales, as intuitively expected.

### F. Conclusions

Driven by the need to understand crowding effects in protein mixtures as present for example in the living cell, we have embarked on a systematic test of the application of simple mixing rules in order to reproduce a number of key properties. We focus, in particular, on macroscopic quantities such as the location of phase boundaries for liquid–liquid phase separation, the osmotic compressibility and the zero shear viscosity, properties on mesoscopic length scales such as the gradient diffusion coefficient, and finally structural and dynamic quantities on local length scales such as the static structure factor and the (local) short-time collective diffusion coefficient. There is no applicable theory capable of incorporating different sizes, interaction potentials, and anisotropy in shape and interactions common for crowded protein mixtures. While one could of course use computer simulations, the resulting computational costs still become unaffordable, in particular, when aiming to reproduce dynamic properties on large length and time scales. Therefore, we have defined different mixing rules for these properties that are all based on the same underlying principle: There is no specific interaction between different species other than excluded volume effects, and we estimate the measured quantities from a weighted sum of the properties of the individual proteins in crowded solutions at an effective volume fraction that takes into account the accessible volume due to the presence of other protein species.

Despite their simplicity, we obtain rather good agreement with experimental data on macroscopic and mesoscopic length scales, i.e., phase behavior, osmotic compressibility, zero shear viscosity, and gradient diffusion. It is only at low volume fractions and low temperatures close to the binodal for LLPS that deviations become quite significant. The agreement is less convincing at intermediate length scales of order the protein size of the larger protein *α*-crystallin, where the lack of a mutual attraction between *γ_B_* and *α*-crystallins results in significant disagreement between calculated and measured values for both *S*(*q*) and *D*(*q*). At even shorter length scales of the size of the smaller protein *γ_B_* crystallin, the finding is mixed, with very good agreement for *S*(*q*), but significant deviations for *D*(*q*). This clearly shows the sensitivity of the local short-time collective diffusion coefficient on patchy attractions that lead to the formation of transient clusters with life times comparable or larger to the decay time probed with NSE.^19^ This is quite in contrast to the mesoscopic gradient diffusion coefficient measured with DLS, where characteristic relaxation times are long compared to the cluster lifetime, and where thus the mixing rule relying on weighted individual properties become quite powerful.

While our approach will require further validation with more complex mixtures, the success of such simple rules for the present system of a binary protein solution, where the individual proteins exhibit vastly different solution properties, appears promising for future applications in real life systems such as cells or other highly crowded protein mixtures.

## IV. METHODS

### A. Sample preparation

Eye lens proteins were extracted from calf eye lenses and purified according to previous work.^25,51^ In brief, *α* crystallin was extracted from the cortical extract solution via size-exclusion chromatography (SEC, Superdex 200 column), using a 52.4 mM phosphate buffer at pH 7.1 in H_2_O. The solvent of the collected fractions was exchanged with a D_2_O phosphate buffer with the addition of 20 mM of 1,4-dithiothreitol (DTT, Sigma-Aldrich, SE), 1 mM of ethylenediaminetetraacetic acid (EDTA, Sigma-Aldrich, SE) and 0.02wt. % sodium azide (Sigma-Aldrich, SE), pH 7.1. Finally, the new solution was concentrated via Amicon Ultra centrifugal filters of 10 kDa (Sigma-Aldrich, SE). The concentration determination was achieved via UV absorption spectroscopy measurements at λ= 280 nm and the specific absorption coefficient $E_{\alpha ,1\;\text{cm}}^{0.1\%,280\;\text{nm}}=$ 0.845 $\text{mL}\cdot {\text{mg}}^{-1}\cdot \text{cm}$.^51^ We converted the weight concentrations to volume fractions, according to the relation ϕ=c·ν, where c is the concentration in mg/mL and *ν* is the voluminosity of the protein. For *α* crystallin, ν= 1.7 mL/g.^24^

For *γ_B_* crystallin, the filtered nuclear extract was purified through SEC (same as above), using a 275 mM sodium acetate buffer (Sigma-Aldrich, SE) in H_2_O, pH 4.5 as a mobile phase. The collected fractions were then further purified via ion-exchange chromatography (IEX, SP Sepharose Fast Flow, GE Healthcare, USA), using a sodium acetate buffer in H_2_O, pH 4.8 with a 0–325 mM sodium chloride (Sigma-Aldrich, SE) gradient. This allowed the collection of the pure *γ_B_* protein according to its isoelectric point (pI).^63^ The following steps (solvent exchange, sample concentration, protein concentration determination using a specific absorption coefficient $E_{\gamma_{B},1\;\text{cm}}^{0.1\%,280\;\text{nm}}=$ 2.18 mL mg^−1^ cm, and conversion to volume fractions) were achieved as described above for *α*-crystallin. In this case, the voluminosity of *γ_B_* corresponds to ν= 0.71 mL/g.^46^

We investigated $\alpha :\gamma_{B}$ mixing ratios of 3:1, 1:1, and 1:3 per volume for the phase diagram, SLS, SAXS, and NSE, while we focused on the 1:1 mixing ratio for DLS and microrheology.

### B. Static and dynamic light scattering

Static light scattering (SLS) experiments and transmission measurements for the determination of *S*(0) were performed on a homebuilt multi-angle light scattering (MA-DLS) instrument. This spectrometer not only allows for suppression of multiple scattering using the 3D cross correlation technology, but has the additional advantage of simultaneous measurements at 4 scattering angles *θ* separated by 30°. The q-range covered by LS is 0.003–0.03 nm^−1^.^64^ Additional dynamic (DLS) and static (SLS) light scattering measurements were performed with a goniometer light scattering setup (3D LS Spectrometer, LS Instruments, AG), implemented with a 3D cross correlation unit to suppress contributions from multiple scattering.^65,66^ Unless stated, all measurements were performed at a *θ* range from 30° to 130° (steps of 5°), corresponding to a scattering vector range q=(4πn/λ) sin(θ/2)≈ 0.006–0.023 nm^−1^.

For DLS, intensity auto-correlation functions $g_{2}(q,\tilde{t})-1$ were converted to the intermediate scattering functions $g_{1}(q,\tilde{t})$ via the Siegert relation, $g_{1}=\sqrt{\frac{1}{\tilde{\beta }}(g_{2}(q,\tilde{t})-1)}$, where $\tilde{t}$ is the lag-time and $\tilde{\beta }$ is the spatial coherence factor, determined by the intercept of $g_{2}(q,\tilde{t})-1$. The intermediate scattering functions from *q*-dependent DLS were then described by fitting the initial part of the correlation function $g_{1}(q,\tilde{t})$ until it has decayed to 80% of its initial amplitude, using a single exponential function

$$g_{1}(q,\tilde{t})=\text{exp}(-\Gamma \tilde{t}),$$ (16)

where Γ is the relaxation rate and corresponds to the inverse of the relaxation time *τ*. The collective diffusion coefficients *D_c_* were then determined according to the relation $D_{c}=\Gamma /q^{2}$, which corresponds to the slope of the linear fit of Γ vs *q*^2^.

### C. Microrheology

Microrheology was performed via DLS^61^ and multiple particle tracking (MPT) using confocal laser scanning microscopy (CLSM).^67^ Tracer particles were prepared according to,^68^ using polystyrene particles stabilized with covalently bound 20 kDa poly(ethylene) glycol chains. For DLS-based microrheology, we use a particle diameter *d* = 300 nm. On the other hand, for CLMS-based MPT we rely on fluorescent-labeled polystyrene particles, *d* = 1 *μ*m. The colloidal stability of the tracer particles was tested *a priori* according to previous work.^61,68^

For DLS measurements, a volume of 1 *μ*L of the tracer particle solution (concentration ≈ 2 wt. %, number density ≈ 10^12^ particles · cm^−3^) was added to 100 *μ*l of protein solution. All DLS experiments were carried out at a single θ= 90° at T = 25 and 35 °C. The addition of tracer particles to the protein solutions results in a double-step relaxation process in the $g_{2}(q,\tilde{t})-1$ function, originating from the scattering contributions from the proteins and the tracer particles.

In our measurements, the slow relaxation mode resulting from the tracer particles (i.e., at large $\tilde{t}$) has an amplitude that is > 95% of the total $g_{2}(q,\tilde{t})-B$ function, making the additional fast relaxation contribution from the proteins negligible.^61^

The auto-correlation functions were described with a double exponential function, using an iterative non-linear fitting procedure based on Ref. 69:

$$g_{2}(q,\tilde{t})=B+\tilde{\beta }{\left\{A\cdot \;\text{exp}(-\Gamma_{1}\tilde{t})+(1-A)\cdot \;\text{exp}(-\Gamma_{2}\tilde{t})\right\}}^{2},$$ (17)

where *B* is the baseline, $\tilde{\beta }$ is the spatial coherence factor, *A* is the amplitude of the first relaxation mode $\Gamma_{1}$, i.e., the protein contribution, and $\Gamma_{2}$ is the decay from the tracer particle contribution. The diffusivity of the tracer particle in the protein sample was then calculated as $D_{\text{Sample}}=\Gamma q^{2}$. Using the Stokes–Einstein relation, we then calculated the relative viscosity (*η_r_*) value,

$$\eta_{r}=\frac{\eta_{\;\text{Sample}}}{\eta_{\;\text{Ref}}}=\frac{D_{\;\text{Ref}}}{D_{\;\text{Sample}}},$$ (18)

where $\eta_{\;\text{Sample}}$ corresponds to the zero shear viscosity of the sample and $D_{\;\text{Ref}}$ to the diffusion coefficient of the tracer particle dispersed in pure solvent with the solvent viscosity $\eta_{\;\text{Ref}}$.

For MPT, a volume of 1 *μ*L of the stock solution of the fluorescent particles ( d=1 μm (ThermoFisher, F8820), concentration ≈ 2 wt. %) was added to 100 *μ*l of protein solution. 5 *μ*l protein solution with particles were transferred in a hermetically closed microscope glass slide. After their preparation, the samples were thermally equilibrated (T = 25 and 35 °C) in a Leica SP5 confocal laser scanning microscope equipped with a temperature chamber. Videos of the samples were performed by acquiring 6000 frames, with a rate of 0.13 frame s^−1^ in a sample depth of 10 *μ*m (i.e., distance from the upper glass slide). Videos were then processed via IDL software (L3 Harris Geospatial) and dedicated scripts.^67,70^ The mean square displacement (MSD) and, subsequently, the diffusion coefficient was calculated via the so-called Van Hove self-correlation function.^71–73^ Finally, the viscosity was obtained via the Stokes–Einstein relation and normalized by the viscosity of D_2_O at the given temperature.^74^ In order to reach high ϕ and determine the arrest transition of the sample, we rely on the procedure described in a previous work.^67^ Here, the sample is left to evaporate for a controlled amount of time (from ≈ 1 to 15 min) before closing it hermetically. The difference between the weight of the sample at the moment of its transfer to the glass slide and the weight after the solvent evaporation, allow the determination of the final volume fraction.

The motivation for MPT-based microrheology measurements in addition to the DLS-based microrheology is twofold. First, with MPT microrheology, we bypass the contribution of critical fluctuations around the critical volume fraction that create interference with the signal of the tracer particles in DLS. Second, MPT allows to determine the point where the glass transition occurs.^67^

### D. Small angle x-ray scattering

Small angle x-ray scattering (SAXS) measurements were performed with a pinhole camera system (Ganesha 300 XL, SAXSLAB) equipped with a high brillance microfocus sealed tube and thermostated capillary stage. The accessible *q*-range for these measurements was from 0.06 ${\text{nm}}^{-1}\leq q\leq 6$ nm^−1^. All measurements were corrected for background radiation, transmission, buffer, capillary, and renormalized for the protein concentration.

### E. Neutron spin echo spectroscopy

Neutron spin echo (NSE) experiments were performed at the J-NSE instrument^75^ at the Heinz Maier-Leibnitz Zentrum (Munich, DE). The probed q-range spanned from 0.1 to 2.3 nm^−1^ and a Fourier time ( ${\tilde{t}}_{i}$) between 0.03 and 140 ns. Intermediate scattering functions for NSE were fitted with a single exponential decay for the initial decay (*t* < 50 ns): $I(q,{\tilde{t}}_{i})=\text{exp}(-\Gamma {\tilde{t}}_{i})$ and the diffusion coefficients were calculated via $D_{\text{NSE}}=\Gamma q^{2}$. For pure *α* and *γ_B_* crystallin solutions, the diffusion coefficients were taken from Ref. 19 and interpolated to match the right volume fraction range (for details see supplementary material).

## SUPPLEMENTARY MATERIAL

See the supplementary material for the following additional information: Examples for microrheology results from 3D-DLS and MPT-based measurements using confocal laser scanning microscopy (Fig. S1); a table with the values of the parameters used for the parameterization of the relative viscosities of the pure crystallin solutions (Table S1); concentration and temperature dependence of the gradient diffusion coefficient and the osmotic compressibility of *γ_B_* crystallin determined by DLS and SLS (Fig. S2); visualization of compilation of *γ_B_* crystallin structure factors as a function of volume fraction and temperature (Fig. S3); interpolation of diffusion coefficients from NSE as a function of volume fraction for the pure crystallin solutions (Fig. S4); *q*-dependent diffusion coefficients (Fig. S5), and examples of intermediate scattering functions (Fig. S6) from NSE for different binary crystallin mixtures.

## Data Availability

The data that support the findings of this study are available from the corresponding author upon reasonable request.
